# Supramolecular Control of Singlet Oxygen Generation

**DOI:** 10.3390/molecules26092673

**Published:** 2021-05-02

**Authors:** Akshay Kashyap, Elamparuthi Ramasamy, Vijayakumar Ramalingam, Mahesh Pattabiraman

**Affiliations:** 1 Department of Chemistry, University of Nebraska Kearney, Kearney, NE 68849, USA; akskashyap65@gmail.com; 2 Department of Chemistry and Biochemistry, The University of Texas at Arlington, Arlington, TX 76019, USA; elamparuthi.ramasamy@uta.edu; 3 Department of Biology and Chemistry, SUNY Polytechnic Institute, Utica, NY 13502, USA; ramaliv@sunypoly.edu

**Keywords:** singlet oxygen, supramolecular chemistry, cavitands, cucurbituril, cyclodextrin, calixarene, singlet oxygen generation (SOG), photosensitizer (PS), oxidation, photodynamic therapy (PDT), near IR (NIR)

## Abstract

Singlet oxygen (^1^O_2_) is the excited state electronic isomer and a reactive form of molecular oxygen, which is most efficiently produced through the photosensitized excitation of ambient triplet oxygen. Photochemical singlet oxygen generation (SOG) has received tremendous attention historically, both for its practical application as well as for the fundamental aspects of its reactivity. Applications of singlet oxygen in medicine, wastewater treatment, microbial disinfection, and synthetic chemistry are the direct results of active past research into this reaction. Such advancements were achieved through design factors focused predominantly on the photosensitizer (PS), whose photoactivity is relegated to self-regulated structure and energetics in ground and excited states. However, the relatively new supramolecular approach of dictating molecular structure through non-bonding interactions has allowed photochemists to render otherwise inactive or less effective PSs as efficient ^1^O_2_ generators. This concise and first of its kind review aims to compile progress in SOG research achieved through supramolecular photochemistry in an effort to serve as a reference for future research in this direction. The aim of this review is to highlight the value in the supramolecular photochemistry approach to tapping the unexploited technological potential within this historic reaction.

## 1. Introduction

Singlet oxygen (^1^O_2_) is the common name of an electronically excited state of molecular oxygen, which is less stable than the ubiquitous triplet molecular oxygen (^3^O_2_) in its ground state that is found (and referred to) as ambient oxygen [1,2]. Though chemists understood that the combination of light and organic dyes generated a highly reactive oxygen species in the 1930s, a clear picture of its electronic state and intermediary nature did not emerge until the late 1960s [3,4]. As the excited state electronic isomer of ambient oxygen, ^1^O_2_ possesses higher oxidative power and reactivity than ambient oxygen [5]. These features confer a great deal of biological and chemical significance on this reactive oxygen species. Singlet oxygen can be produced as a product in a (ground state) reaction between sodium hypochlorite and hydrogen peroxide [6]. It can also be generated through direct laser excitation of ^3^O_2_ at ~1064 nm [7]. However, the most convenient way of accessing this species is through sensitization, wherein a photosensitizer in the excited triplet state relaxes to its ground state by triplet energy transfer to ambient oxygen, resulting in ^3^O_2_ → ^1^O_2_ [8].

**Figure 1:**
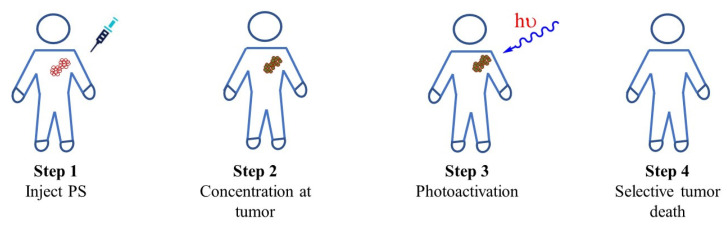
Biological and therapeutic applications of singlet oxygen.

The biological relevance of this molecular species can be deduced from the works published on its role as reactive oxygen species (ROS) in botany [9], atmospheric chemistry [10], cellular signaling [11], and physiopathology [12]. In medicine, photo-dynamic therapy (PDT, Figure 1) is a singlet oxygen-derived treatment modality that utilizes its cytotoxicity to achieve selective apoptosis or necrosis through its localized generation [13,14]. Due to its target specificity and minimal invasiveness, PDT is the preferred mode of treatment for conditions such as psoriasis [15,16], certain forms of cancers (skin, bladder, lung) [17,18,19], macular degeneration [20]. As a reactive oxygen species (ROS), its biological oxidizing propensity holds promise as a disinfection technology to neutralize bacteria and viruses [21,22].

**Figure 2:**
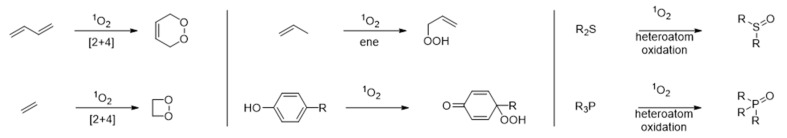
Chemical reactions affected by singlet oxygen.

The increased oxidative power and electronic configuration of ^1^O_2_ has given rise to its use as a synthetic tool (Figure 2) to access numerous organic intermediates that are not easily achieved through classical organic transformations [23,24,25]. Reactions such as singlet-ene [26,27], cycloaddition (4 + 2 and 2 + 2) [28], and heteroatom oxygenations [29,30] have been useful in fine chemical and large-scale synthesis of numerous natural products and drugs (include pictures of reactions). A recently fast-growing interdisciplinary area of research involving singlet oxygenation is designing functional systems such as self-cleaning [31] and disinfecting materials [22,32], and lasers for solar energy conversion [33].

As outlined above, there has not been any shortage of scientific exploration into this ROS or its central in role in modern technology. It is predicted that there will be a growing demand for newer inventions for medicinal, pharmacological, and chemical applications based on this chemical entity. While much is known about the electronic nature of singlet oxygen, its excited state dynamics, and physical properties, there are also limits to its usefulness owing to theoretical and practical impediments associated with its uncontrolled reactivity, medium-influenced deactivation, and deactivation pathways [34]. Chemists have developed several strategies to overcome these disadvantages and have made tremendous progress in recent years in developing newer technologies based on this reaction [35,36,37]. One such approach is the use of non-covalent interactions to control the photosensitizer to modify singlet oxygen generation and its reactivity: the supramolecular approach. There are several advantages to the supramolecular approach over others, such as simplicity, the reduced need for chemical alterations of the PS, greater photodynamic control, etc.

In the broad swath of singlet oxygen literature, the supramolecular approach to SOG is a relatively less-explored endeavor. To our knowledge, there is no such review that covers the research activity in this direction; however, there is a sufficient quantity of individual research articles and scientific novelty in those works to be represented in a review, which is the aim of this effort. This review will compile research activity that has utilized supramolecular interactions to control singlet oxygen generation and its reactivity. Consistent with the majority of the published literature in this area, which has used cavitands for controlling PS photochemistry, this review will predominantly contain instances of SOG influenced by cavitands (a subset of supramolecular chemistry) with few examples outside the host–guest chemistry realm.

## 2. Singlet Oxygen Background

**Figure 3:**
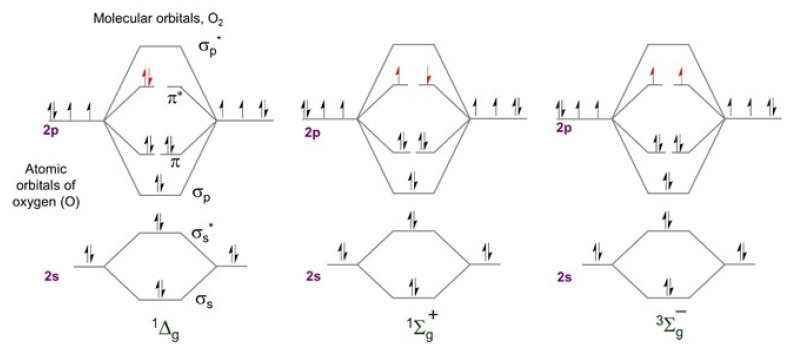
Molecular orbital energy diagram for the two excited singlet states of oxygen (left, and middle) and triplet ground state.

**Figure 4:**
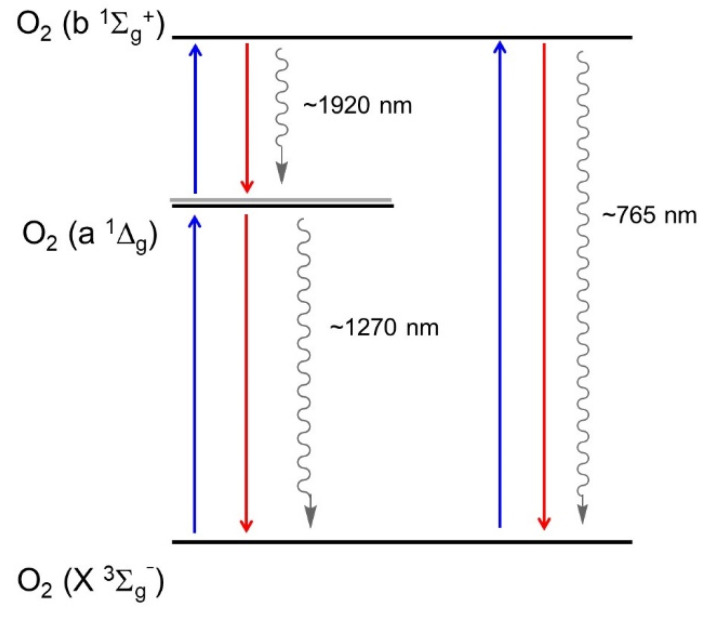
Relative arrangement of electronic states of molecular oxygen.

**Figure 5:**
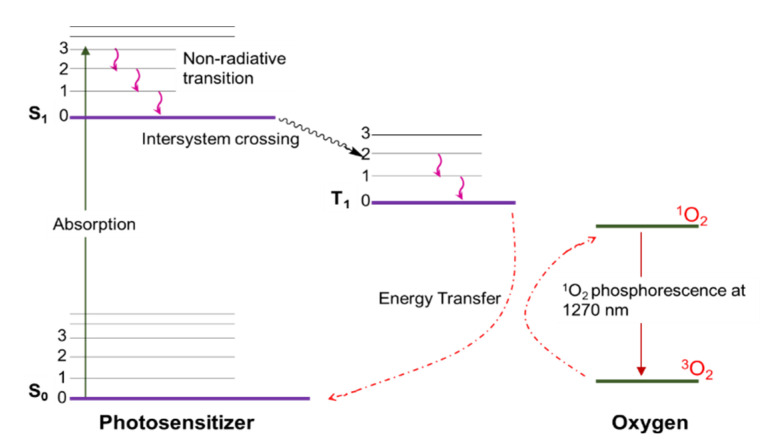
Jablonski diagram representing singlet oxygen generation through triplet energy transfer from photosensitizer.

Singlet oxygen is the common name of the electronically excited state of molecular oxygen, which is less stable than molecular oxygen in its electronic ground state (Figure 3). Molecular oxygen in its ground state contains two unpaired electrons. This is due to two degenerate orbitals in its HOMO (^3^∑_g_^−^, Figure 3, right). The vertical alignment of three states in order of increasing energy is presented as a diagram in Figure 4. Two excited singlet (electron-coupled) states exist for oxygen—^1^∑^+^_g_ and ^1^∆_g_—that are higher in energy by 150 and 95 kJ/mol, respectively [38,39]. Under typical experimental conditions, the ^1^∑_g_^+^ (upper excited) state rapidly relaxes to the lower energy ^1^∆_g_ state; therefore, the vast majority of the photophysicochemical phenomena of singlet oxygen are observed from the ^1^∆_g_ state. Singlet oxygen is typically generated via energy transfer from the excited state of a photosensitizer to the oxygen molecule (Figure 5). Photoexcitation of a PS molecule, which possesses high ISC rates, engages in efficient spin-coupled triplet energy transfer (T_1,PS_ → S_0,PS_) to triplet oxygen (T_1,O2_ → S_1,O2_). Singlet oxygen thus generated is a much more reactive form of oxygen, engaging in redox chemistry or weak phosphorescence in the infrared region.

In photosensitized SOG, a sensitizer (typically a dye molecule) absorbs light in the visual spectrum, resulting in its excitation to its first singlet state (S_0,PS_ → S_1,PS_), followed by rapid intersystem crossing to its first triplet state (S_1,PS_ → T_1,PS_). In this stage the excited PS and molecular oxygen engage in efficient spin-coupled triplet energy transfer (T_1,PS_ → S_0,PS_)//(T_1,O2_ → S_1,O2_). Later advancements in laser technology leading to the advent of powerful YAG lasers allowed direct excitation of ^3^∑_g_^−^ state to ^1^∑_g_^+^ when excited at 1064 nm. Spin-allowed transition (singlet to singlet) and energetic proximity between the two excited states results in rapid ^1^∑_g_^+^ → ^1^∆_g_ relaxation, which precludes any physicochemical processes this species could undergo. However, photosensitized generation is the most efficient, practical, and convenient method for producing singlet oxygen.

### 2.1. Stability, Detection and Quantification of Singlet Oxygen

Once generated, the prevalence of singlet oxygen in the medium can be observed directly or indirectly (vide infra). Singlet oxygen in the ^1^∑_g_^+^ state is a very short-lived species with estimated lifetimes in the range of 10^−9^ to 10^−11^ s in solution; the lifetimes are higher in gas phase due to lack of solvent-assisted non-radiative decay. On the other hand, ^1^∆_g_ is significantly long-lived as ^1^∆_g_ → ^3^∑_g_^−^ is spin-forbidden, resulting in its metastability. This bears out in the measured lifetimes of this species ranging in 10^−3^ to 10^−6^ s in solution and typically in milliseconds in gas phase, and at times even extending into minutes [40].

Direct observation is achieved through spectroscopic monitoring of its radiative deactivation pathway for the ^1^∆_g_ → ^3^∑_g_^−^ transition, which results in its phosphorescence at 1268.7 nm. The intensity of phosphorescence of singlet oxygen registered in a calibrated optical device is taken as a direct quantitative measure of its generation, which is useful in calculating concentration-dependent quantities such as quantum yield. The unique NIR phosphorescence of singlet oxygen is unambiguous, convenient, and direct; as a result, its direct observation is highly advantageous whenever possible. However, this method often suffers from weak signal intensity due to competing non-radiative pathways that are generally more efficient.

Indirect detection refers to the estimation of SOG based on its reactivity with a chemical agent, whose conversion is monitored through spectroscopy, spectrometry, or chromatography. Commonly used reactions in this regard include oxygenation of tetramethyl piperidine (TEMP) to form TEMPO, cycloaddition with anthracene followed by rearrangement to yield anthraquinone, alcohols resulting from singlet oxygen-ene reaction, etc. [41]. The spectroscopic signals of the product in comparison to the reactant are used to quantify SOG. Examples include following absorption changes for the product or the reactant, emission intensity of fluorophores, EPR signals of radical products such as TEMPO, ratiometric analysis of reactant/product signals, etc. Both methods—direct detection vs. probe reactions—offer advantages and limitations, and typically more than one option is used to reliably quantify singlet oxygen production.

### 2.2. Sensitized Singlet Oxygen Generation

Energy transfer between a photosensitizer (PS) in excited triplet state and ^3^Σ_g−_, to excite the latter to ^1^D_g_ or ^1^S_g_^+^ is referred to as sensitized singlet oxygen generation (SOG). Photosensitizers that have a triplet state higher than the molecular oxygen’s lower excited states (E_T,sens_ > 95 kJ/mol; E_a_ − E_X_ = 94.3 kJ/mol; E_b_ − E_X_ = 157 kJ/mol; reference Figure 4) are theoretically capable of serving as a sensitizer. However, practically useful sensitizers should possess additional features such as high energy-transfer efficiency to ^3^Σ_g−_, resulting in high quantum yield of singlet oxygen generation (^1^Φ_O2_). This requires that the PS possesses the ability to maintain long triplet lifetimes as this increases the probability of energy transfer (and therefore greater ^1^Φ_O2_).

### 2.3. Host–Guest Chemistry and Nano-Containers

Encapsulation of photoactive guest molecules such as photosensitizers within macrocyclic hosts has been a very efficient and simple approach to directing excited state molecular behavior [42,43]. The field of supramolecular photochemistry is dedicated to this endeavor. The advantage of the host–guest approach over others derives from the convenience due to reversibility, and the qualitative predictability of the complex structure. Formation of host–guest inclusion complex is typically driven by two types of interactions: attractive host–guest and/or solvophobic interactions. Combination of host–guest, guest–guest, host–solvent, and guest–solvent interactions have been skillfully employed by chemists to precisely control excited state behavior.

**Figure 6:**
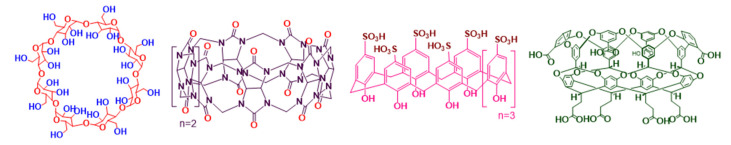
Chemical structures of common molecular cavitands used to control excited-stated chemistry of organic molecules.

**Figure 7:**
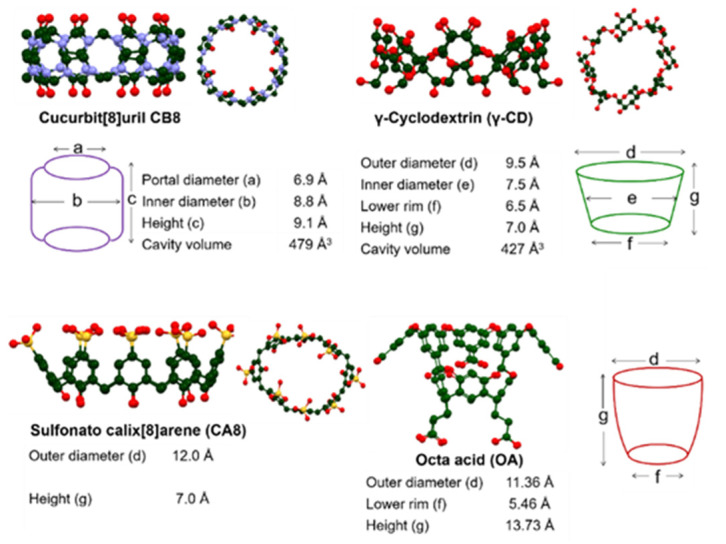
Representations of cavitands frequently used in controlling singlet oxygen generation efforts. Color coding: red (oxygen), blue (nitrogen), green (carbon), yellow (sulfur). Hydrogens are omitted for clarity.

Several hosts are currently available for supramolecular chemists, although a few macrocycles have been employed more frequently than others. Cyclodextrins (CDs) [44,45] and cucurbiturils (CBs) [46,47] are arguably the most commonly utilized organic hosts due to their supramolecular versatility, easy synthetic accessibility, and aqueous amenability; this is also true in the case of singlet oxygen generation efforts. Calixarenes (CAs) [48] are the next most commonly utilized host, though to a much lower extent than the former. Each family of the aforementioned macrocycles possesses oligomers that are of comparable dimensions to each other; as a result they affect similar photochemical outcomes in many instances. At the same time, differences in their chemical functionalities, and therefore different modes of interactions, have led to markedly different photochemistry as well. Octa acid (OA) [49] is a relatively newly synthesized host with unique host characteristics, which has given rise to previously unobserved inclusion dynamics and, therefore, supramolecular photochemistry. The chemical structures of common hosts are provided in Figure 6, and their dimensions and comparative 3-D structures rendered through molecular modeling are provided in Figure 7.

## 3. Supramolecular Approaches to Singlet Oxygen Generation

**Figure 8:**
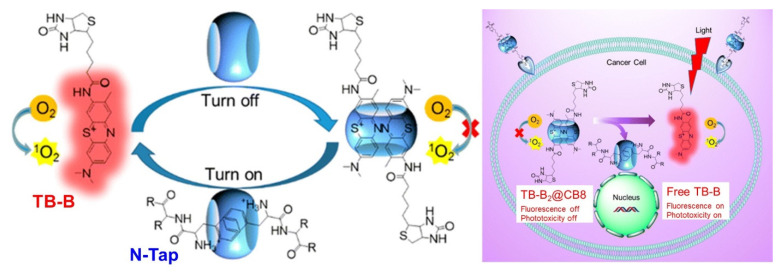
(**Left**) Cavitand-based activatable photosensitizer (aPS) assembly for singlet oxygen generation and simultaneous imaging. (**Right**) Utility of aPS for simultaneous cellular PDT and imaging. Image used with permission from original work [50].

A cavitand-regulated activatable photosensitizer with a dual role as singlet oxygen generator (SOG) and fluorescent imager was reported by Wang et al. (Figure 8) [50]. Activatable photosensitizers (aPSs) are photodynamic therapy (PDT) agents that possess the ability to simultaneously image cancer location and its selective ablation. Traditional synthetic approaches to designing aPSs are inefficient and tedious due to the need for combining various modules (photo-, physio-, and biochemical) covalently. An efficient approach to the construction of a ter-modal system can be achieved through host–guest inclusion based on supramolecular interactions. The system reported by Wang is based on the host−guest interaction between the biotinylated toluidine blue (TB-B, Figure 8) and CB8 to form ternary TB-B_2_@CB8 complex. This is a three-component system with the biotin unit acting as cell-receptor anchoring unit, TB-B as singlet oxygen generator and fluorophore, and CB8 as function regulator, as depicted in Figure 8 (left).

**Figure 9:**
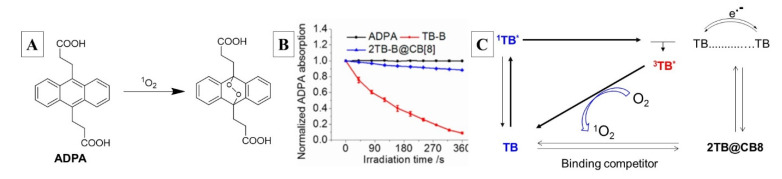
(**A**) Oxidation reaction used to monitor singlet oxygen generation. (**B**) Trends in singlet oxygen generation based on absorbance of ADPA alone (black), and ADPA with TB-B (on state, red) and ADPA, TB-B, CB8 and binding competitor (off state, blue). (**C**) Mechanism of energy and electron transfer pathways for on/off state. Images used with permission from published work [50].

TB-B is a phenothiazinium dye (acting as a PS) with dual activity as an efficient SOG and fluorophore. As a free, uncomplexed dye, TB-B generates singlet oxygen, which is monitored through the endoperoxidation of anthracene 9,10-dipropionic acid (ADPA, Figure 9A,B). As a luminophore, free TB-B also possesses strong fluorescence at ~650 nm. In the presence of added CB8, the dye self-assembles into a ternary complex (TB-B_2_@CB8) with head-to-tail guest arrangement owing to steric interaction between the bulky biotin side arms. With CB8 acting as a physical barrier, the SOG ability of TB-B is suspended; as two dyes are included within the cavity, TB-B also loses its ability to fluoresce due to self-quenching presumably because of electron transfer (Figure 9C). Releasing complexed TB-B through competitive guests known to have very high affinity for CB8, such as N-terminated aromatic peptides, restores its photophysical activity. The utility of such a system for simultaneous tumor oblation and imaging is presented in Figure 8 (right): the biotin arm anchors the photo-inactive ternary complex (TB-B_2_@CB8) onto the cell-surface receptor, which is then transported into the cell where intracellular N-terminated aromatic peptides (N-Tap, Figure 8) release TB-B, restoring its photodynamic activity and fluorescence. Monitoring of singlet oxygen generation through absorbance change for ADPA and energy/electron transfer pathways is summarized in Figure 9.

**Figure 10:**
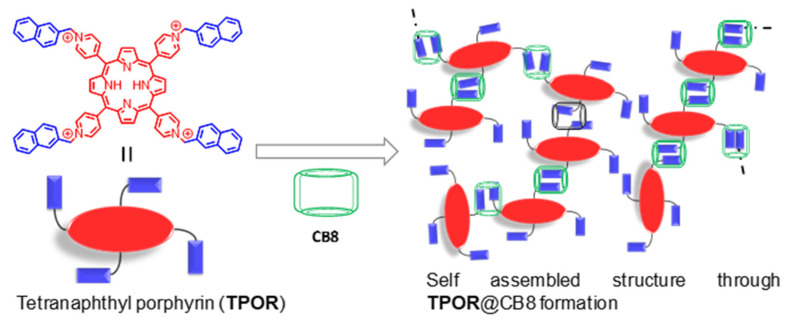
Cucurbituril-mediated enhancement of singlet oxygen generation efficiency of naphthyl TPOR: Structure of TPOR and complexation of CB to form TPOR-CB8 network [51].

**Figure 11:**
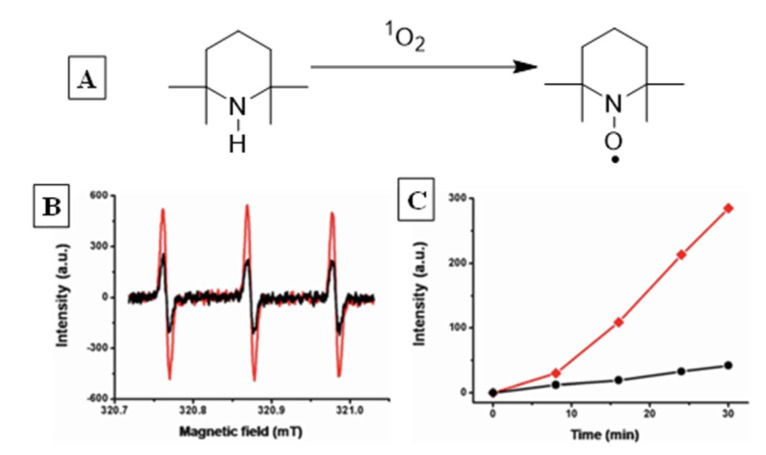
(**A**) Reaction used for monitoring SOG by TPOR@CB8 self-assembled polymer. (**B**) TEMPO EPR signal for uncomplexed (black) and complexed (red) TPOR. (**C**) Plot of time-dependent change in TPOR oxidation. Images used with permission from published work [51].

The role of CB in a self-assembled polymeric network capable of generating singlet oxygen was demonstrated by Liu et al. [51]. Hyperbranched supramolecular polymers were obtained by mixing a tetranaphthyl-substituted porphyrin (TPOR, Figure 10) derivative and CB8 in aqueous solution, which was driven by host–guest interactions. The formation of a supramolecular polymeric structure can cause disruption of the porphyrin aggregation, thus leading to enhancement of their singlet oxygen generation (SOG) efficiency. Unlike the previous example (Figure 9) where complexation-induced suspension in SOG efficiency was observed, the TPOR polymeric network is photo-inactive when uncomplexed and generates singlet oxygen upon inclusion and polymer formation. This is because porphyrins are poor SOGs in general, as they remain aggregated due to their large, rigid, planar structures, which leads to aggregation-induced photo-deactivation. Affinity of CB8 for cationic guests disaggregates the TPOR units, while its ability to include two guests simultaneously results in formation of an extended …TPOR..CB..TPOR..CB..TPOR… network. The SOG efficiency of the polymer was monitored through the oxidation of tetramethyl piperidine (TEMP → TEMPO) analyzed with EPR spectroscopy (Figure 11). TEMP is EPR-inactive while the oxidized product is a radical that presents a clear signal indicating the presence of singlet oxygen. The change in EPR-signal-monitored TEMP exposed in the presence and absence of polymeric TPOR shows the latter’s SOG efficiency.

**Figure 12:**
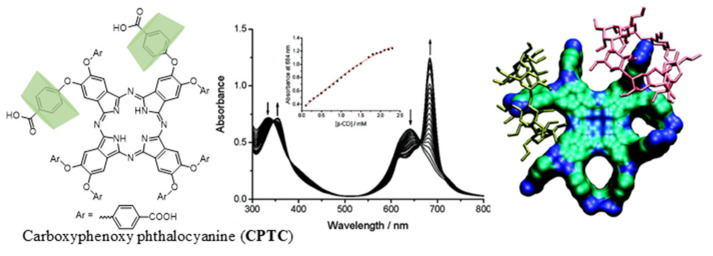
(**Left**) Chemical structure of CPTC and its complexation with host to form CPTC@*β*-CD_2_ complex. (**Middle**) Complexation-induced changes in UV-Vis absorption spectrum. (**Right**) Energy-minimized structure of complex. Image used with permission from published work [52].

Another example of enhanced SOG of an otherwise photoactive dye rendered inactive due to aggregation-induced photo-deactivation was reported by Leng et al. (Figure 12) [52]. Carboxyphenoxy phthalocyanines (CPTCs) were synthesized and tested for their nascent SOG efficiency, which was quite low due to strong tendency for π-stacked aggregation. Disaggregation through complexation using *β*-CD to form a 1:2 (CPTC@*β*-CD) complex showed significant changes in absorption and emission characteristics as well as its SOG efficiency.

The use of CBs in manipulating SOG efficiency of TPOR and its antibacterial efficacy in solid-state was reported by Liu et al. [53]. The previous examples demonstrated the value of cavitands in SOG in homogeneous media; the same in solid-state is even more difficult as there is greater tendency for aggregation among dyes. Employing neutral hosts such as cyclodextrins does not guarantee disaggregation in solid-state as protruded units of the guests can engage in aggregation leading to photochemical deactivation. This tendency for aggregation despite complexation can be avoided if ionic dyes are employed.

**Figure 13:**
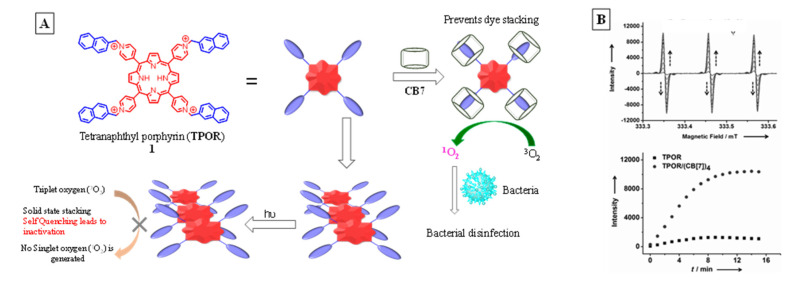
(**A**) Depiction of cucurbituril-TPOR complex capable of generating singlet oxygen for bacterial disinfection. (**B**) Comparison of TPOR’s SOG in the presence (circle) and absence (square) of CB8 as monitored through EPR spectroscopy of TEMP oxidation to TEMPO. Images used with permission from published work [53].

Liu et al. reported lowering of aggregation-induced deactivation upon complexation to CB as shown in Figure 13. The dye, owing to its cationic charge was also well-adsorbed onto Gram-negative bacteria (*Escherichia coli*). This led to strong bacterial disinfection efficacy even in solid-state. The SOG efficiency of the complexed substance was followed by monitoring the oxidation of TEMP in EPR (Figure 13B). Changes in the EPR signal for the free and complexed EPR oxidation of TEMP showed a significantly marked increase in singlet oxygen when the dye was complexed to CB8. Time-dependent studies showed a steady increase in EPR signal for the TPOR@CB7 complex, producing singlet oxygen indicating formation of TEMPO; the plot of EPR intensity for a mixture of photosensitizer, TEMP mixture in the presence and absence of CB7 clearly demonstrated the role of cavitand in manipulating SOG efficiency (Figure 13).

**Figure 14:**
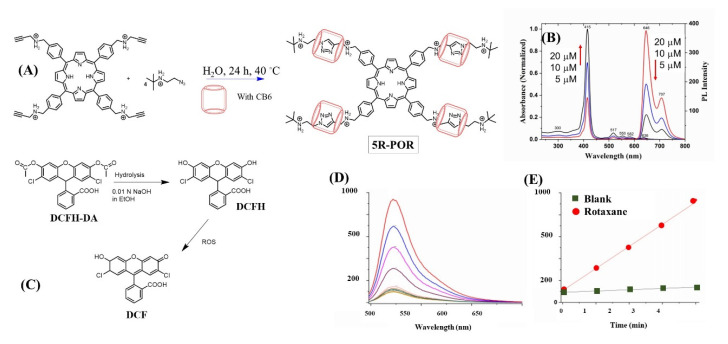
(**A**) Depiction of 5R-POR complex formation for singlet oxygen generation. (**B**) Absorption and emission spectra of the uncomplexed POR. (**C**) Redox reaction with singlet oxygen used to monitor its generation. (**D**,**E**) Emission intensity change and its plot for singlet oxygen monitoring. Images used with permission from published work [54].

A similar approach of enhancing SOG by preventing π-stacking of porphyrins using cucurbiturils was demonstrated by Özkan et al. [54]. In this case, their approach involved synthesizing a stable [5] rotaxane porphyrin (5R-POR) with CB6, which self-generates through catalyzing 1,3-dipolar cycloaddition (click reaction, Figure 14A) between alkyne-substituted porphyrin and an azide-functionalized stopper core. Supramolecular influence on modified photophysics of the sensitizer was clearly evidenced in its concentration-dependent emission studies as an increase in concentration (5 to 20 μM) and showed a clear decrease in emission intensity due to self-quenching (Figure 14B) as the result of aggregation. Fluorescence quantum yield and lifetime measurements were 0.01 and 7.5 ns, respectively, in water at 5 μM. The oxidation reaction of 2,7-dichlorofluorescein (DCF) diacetate with singlet oxygen to produce a highly fluorescent 2,7-dichlorofluorescein was used to monitor SOG efficiency (Figure 14C) wherein singlet oxygen production was proportional to the DCF concentration and, hence, higher fluorescence intensity; comparison of fluorescence of DCF in the presence of rotaxane with that of control (non-rotoxane porphyrin) showed a clear enhancement in SOG (Figure 14D,E). The application of this system in photodynamic disinfection was demonstrated against Gram-positive and Gram-negative bacteria.

**Figure 15:**
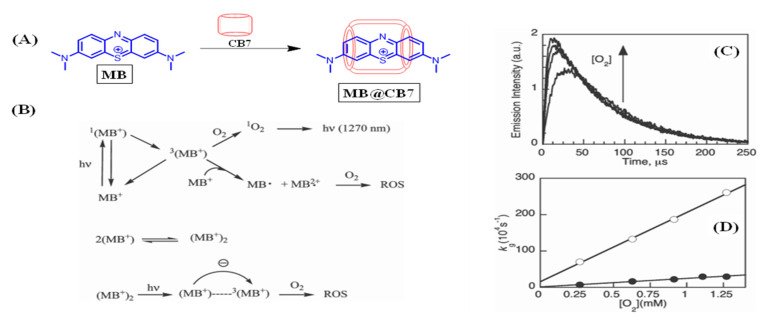
(**A**) Schematic representation of methylene blue complexing to CB7. (**B**) Photo-dynamics of singlet oxygen generation by free and bound MB. (**C**,**D**) Spectral monitoring of singlet oxygen generation and its plotting in the absence (O) or presence (●) of CB7. Images used with permission from published work [55].

Sciano’s group investigated the effect of CB7 on the SOG efficiency of methylene blue (MB, Figure 15, scheme A), a well-known and well-studied dye utilized for this purpose [55]. They studied the temporal characteristics of the excited MB in free and complexed form through laser flash photolysis (LFP) and also monitored the NIR luminescence of ^1^O_2_. It was observed that around 2% of MB remained aggregated in micromolar concentrations (5.8 × 10^−6^ M), and addition of CB7 would have prevented aggregation, thereby resulting in higher SOG efficiency.

However, to the contrary, a slight decrease in NIR luminescence of ^1^O_2_ was observed for MB@CB7 at micromolar concentrations (Figure 15C). Analysis of luminescence decay at 1280 nm resulting from a 308-nm laser excitation showed a mono-exponential decay with lifetimes of ~70 μs for the ^1^O_2_ for both free and complexed dye. However, there was a noticeable lag (delay) in its generation and slightly decreased intensity, while Φ*_SOG_* only decreased slightly (0.44) upon complexation compared to the free dye (0.52). This was deduced to be a kinetic effect, which resulted from the increased energy barrier imposed by an encapsulating cavitand that ground-state oxygen (^3^O_2_) has to overcome to achieve an energy transfer (quenching) from the ^3^MB. This provided an opportunity for mechanistic insight into the singlet oxygen generation processes as quantified by the penetration rate constant of 2 × 10^−8^ M^−1^ S^−1^. This cavitand-imposed barrier effect that manifested as a change in kinetic parameter correlated with decreased ^1^O_2_ quenching rate constant for MB@CB7 (2.6 × 10^−9^ M^−1^ S^−1^ vs. 0.9 × 10^−9^ M^−1^ S^−1^).

**Figure 16:**
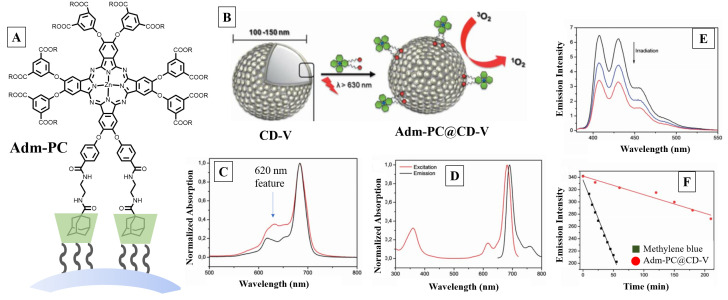
(**A**) Adamantyl phthalocyanine (Adm-PC) bound to the surface cavity of CD-V nanoparticle. (**B**) Formation of CD-V nanoparticle by tethered CD and representation of overall complexed structure. (**C**,**D**) Spectra of free (red) and bound (black) Adm-PC. (**E**,**F**) Monitoring of singlet oxygen generation through spectral changes of ADMADM oxidation and its plot. Images used with permission from published work [56].

The use of cavity-containing vesicles constructed with *β*-cyclodextrin derivatized with lipophilic sidearms to produce singlet oxygen was demonstrated by Voskuhl et al. [56] *β*-CDs derivatized with 7-n-dodecylthiol units on the primary rims and with oligo(ethylene glycol) was used to prepare the cyclodextrin vesicle (CD-V, Figure 16), which resulted in 100 nm unilamellar bilayer structure. The cavities of CDs in the CD-V served as the host to encapsulate the adamantyl sidearm tethered to an asymmetric zinc(II) phthalocyanine dye with adamantyl anchor points (Adm-PC) to function as the sensitizer. Inclusion of the adamantyl sidearm into the *β*-CD cavity resulted in the dye clasped to the vesicle through host–guest inclusion. Disaggregation of the PC structure in vesicles was inferred from the decreased absorption of the shoulder feature at 620 nm (Figure 16C) and the correlation between absorption and excitation spectra (Figure 16C,D).

The surface-immobilized dye was determined to be four times more effective (Φ_∆_ of 0.2 vs. 0.05 for bound vs. free PC) than the free dye for generating singlet oxygen, based on cycloaddition reaction of generated singlet oxygen with ADMADM (9,10-anthracenediyl-bis(methylene)dimalonic acid), which is a 1,9-anthracene derivative and functions similar to ADPA as a probe for singlet oxygen as depicted in Figure 9; comparative study of the same experiment performed with methylene blue yielded quantum yield of 0.5 (Figure 16E,F). Increased SOG efficiency was attributed to the reduction in “inactive aggregates” due to the decrease in aggregation-induced photo-deactivation. This was inferred from the decrease in intensity of the minor absorption band at 620 nm upon CD immobilization.

**Figure 17:**
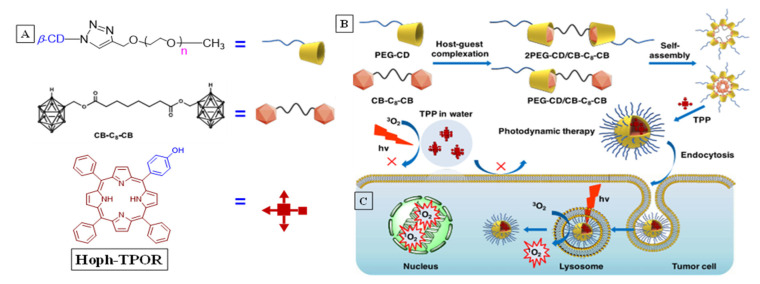
(**A**) Structure of the components of supramolecular assembly. (**B**) Process of the self-assembled micellar nanoparticle. (**C**) Utility of the porphyrin-embedded nanoparticle in tumor cytotoxicity. Used with permission from published work [57].

Xiong et al. combined the host–guest inclusion phenomenon and nanomolecular self-assembly to increase the singlet oxygen efficiency of a porphyrin derivative (5-4-hydoxyphenyl)10,15,20-triphenylporphyrin for intracellular singlet oxygen generation [57]. Favorable binding interaction between carborane (CBn, Figure 17A) and the cavity of polyethylene (PEG)-functionalized cyclodextrin was self-assembled into a nanoparticle (NP, Figure 17B) micellar structure, which contained a well-defined cavity to encapsulate the Hoph-TPOR. The dye encapsulated within the NPs showed a significantly higher degree of singlet oxygen generation compared to the same dyes in aqueous medium without the nanoparticle under control conditions. SOG was monitored using the change in indocyanine green’s (ICG) absorbance as a quantitative measure; reaction between ICG and singlet oxygen resulted in decreased absorbance due to loss of conjugation.

Comparing the time-dependent loss of ICG absorbance at 780 nm for the nanoparticle solution vs. the control groups showed at least twofold increase in SOG. This was established to be due to the prevention of aggregation of TPP within the CD nanoparticle, which increased the efficiency of photosensitization of triplet oxygen; whereas in aqueous media in control groups aggregation-induced non-radiative deactivation was responsible for much lower SOG efficiencies. The nanoparticles also prevented the oxidative bleaching of the dyes, which is often the case when singlet oxygen oxidizes the dye, by preventing physical contact between the two entities.

**Figure 18:**
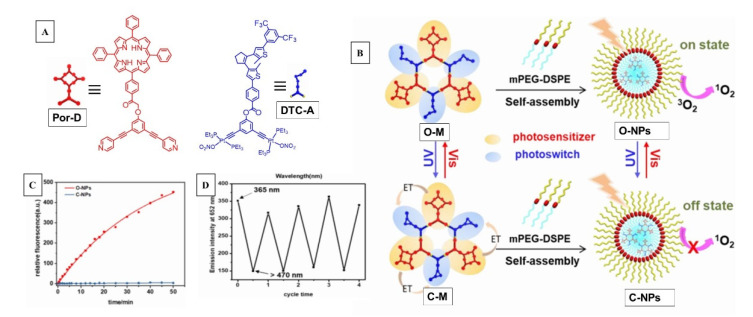
(**A**) Structure of donor and acceptor/switch units in on/off SOG switch. (**B**) Self-assembled structure and their photoreversibility, depiction of assembly embedded within nanoparticle and its SOG. (**C**) Fluorescence response from SOSG assay of the metallacycle embedded within NPs showing high SOG efficiency with DTC-A in open form (red) compared to the closed form (blue). (**D**) Switching between on/off cycle with UV and visible light and their SOG efficiency with SOSG. Images used with permission from published work [58].

**Figure 19:**
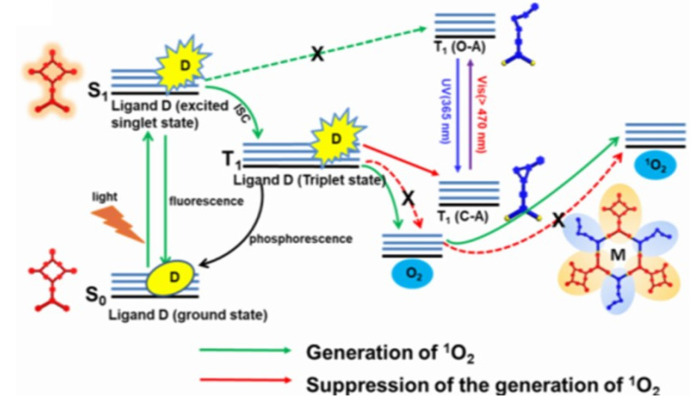
Energy diagram depicting relative energies of Por-D, DTC-A in open and closed forms controlling the on/off mode. Images used with permission from published work [58].

Demonstration of a supramolecular self-assembled and reversing singlet oxygen sensitizer system was reported by Qin et al. [58]. As visually depicted in Figure 18B the dual-stage system assembled through platinum-centered coordination chemistry was composed of a sensitizer porphyrin (Por-D) donor and a dithienylcyclopentene acceptor (DTC-A) that also acts as a photochromic switch that controlled the on/off sensitizing action. In terms of their individual photoactivity, porphyrins are excellent singlet oxygen sensitizers while dithienylcyclopentenes (DTC-A) are known for their reversible, wavelength-dependent ring-opening/closure reactions; they also serve as an energy acceptor from the donor porphyrin in its open form (Figure 19).

In this assembly, the DTC acted to quench the sensitizer in the cyclized (off state) form through Por-D → DTC-A energy transfer as the T_1_ state of DTC in cyclic form is lower than that of Por. Such energy-transfer quenching could not occur in the open form as the T_1_ of open form is higher than that of Por (Figure 19). Due to proximal placement of the functional entities within the well-defined metallacyclic scaffold, ^1^O_2_ generation in the ring-closed form state of the photochromic switch was quenched by photoinduced energy transfer, whereas the generation of ^1^O_2_ in the ring-open form state was activated upon light irradiation. The singlet oxygen generation was monitored by SOSG (singlet oxygen sensor green) fluorescence. The PDT application of this system was achieved by encapsulating this assembly within a nanoparticle system, which showed good tumor toxicity.

**Figure 20:**
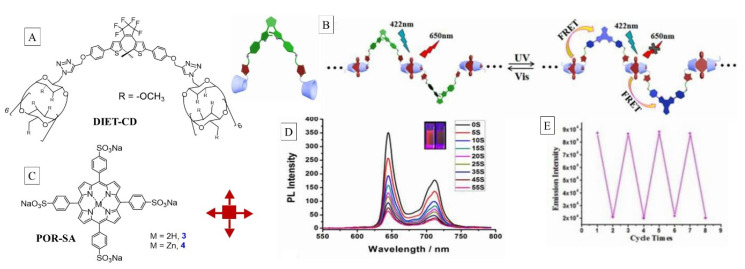
Structure of DIET-CD host (**A**) and porphyrin guest (**C**). (**B**) Photochromic switch dynamics based on the host–guest polymer formation and FRET process and alternating between on/off states with visible/UV lights. (**D**) Change in fluorescence over time when open form is exposed to UV light. (**E**) Switching between the states as monitored through 1283 nm singlet oxygen phosphorescence. Images used with permission from published work [59].

Another supramolecular on/off switch involving a polymeric matrix, which was designed on the donor/acceptor interaction between porphyrin/dithienylethene assembly, was constructed by Liu et al. involving the host–guest inclusion process [59]. This strategy also took advantage of the bifurcated energy-transfer cascade depicted in Figure 19, wherein the photo-dynamics could be directed through optical stimulus. The singlet oxygen sensitizer and energy donor porphyrin (Por-SA) unit was supramolecularly buckled to the DIET-CD acceptor through host/guest inclusion. The dithienylethene moiety could be photochemically reversed between the open and the cyclized using visible and UV light activation (Figure 20). While in the open form, photoexcitation of Por-SA guest at 422 nm resulted in no energy transfer, and hence its fluorescence was observed at 650 nm (fluorescence on state); the unquenched Por-SA is also an excellent SOG sensitizer (on state).

Conversion of the open form to the closed form of DIET-CD through UV light quenched the Por-SA, which was off state for fluorescence and SOG. Irradiation of the mixture with visible light reverted the DET closed form back to the open form, which turned on the SOG production. As can be seen in Figure 20, exposure of the mixture of the open form (SOG on state) to 254 nm UV light resulted in lower emission with time, as the open form converted to closed form. This resulted in Por-SA → DET energy transfer, which also corresponded to decreased SOG. The SOG was monitored using NIR emission of singlet oxygen. The cycling between on/off states was demonstrated by monitoring NIR emission of singlet oxygen with alternating UV and visible light exposure.

**Figure 21:**
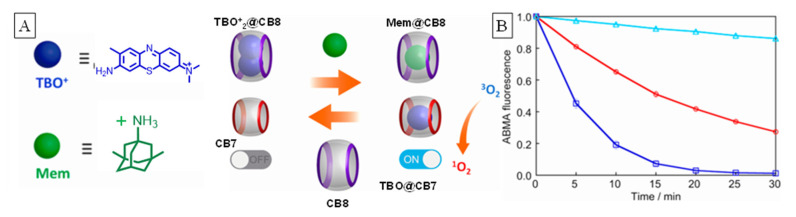
(**A**) The turn on/off of TBO affected through cavitand complexation and competitive guest Mem. (**B**) Fluorescence of ADMADM used to monitor SOG generated when TBO^+^ is exposed to light in the absence of any CBs (red), in the presence of 10.25 eq. of CB7 (dark blue squares) and 8.1 eq. of CB8 (light blue triangle). Images used with permission from published work [60].

An on-off switch based on manipulating the host/guest inclusion equilibrium between two hosts (CB7, CB8) and one PS (toludine blue, TBO^+^) was demonstrated by Robinson-Duggon et al. [60]. The well-known difference in complex stoichiometry (2:1 for CB8 and 1:1 for CB7) and the greater preference of the PS to bind to CB8 over CB7 (1.9 × 10^14^ M^−1^, and 5.5 × 10^6^ M^−1^) were utilized in the design. Dual occupancy of TBO^+^ guest was the off state to aggregation-induced photo-deactivation (Figure 21), whereas singular occupancy within CB7 was the on state for SO generation, especially due to the lack of deactivation and enhanced activity due to cavity-enforced rigidity. The switch started off at an off state with the three components in the mixture with just TBO, CB8 and CB7 in the medium. At this state, stronger affinity of TBO for CB8 over CB7 resulted in a ternary complex formation that self-quenched upon photoexcitation. The PS was activated to the on state when a stronger CB8 guest (memantine, Mem) was added to the medium. The turn-on mechanism involved displacement of the PS by Mem, which resulted in PS@CB7. While in the off state (with CB8) the dye showed a 10% decrease in 9,10-anthracenediyl-bis(methylene)dimalonic acid (ADMADM) fluorescence after 30 min of irradiation, the “on” state reduced its emission intensity to near zero (Figure 20). The free dye showed intermediate activity.

**Figure 22:**
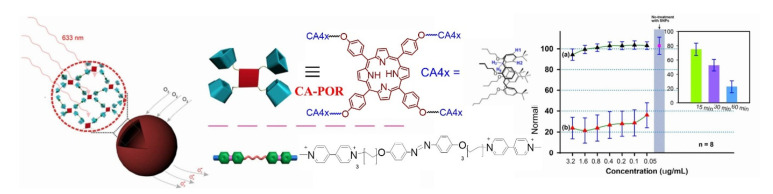
Formation of nanoparticle through supramolecular inclusion of azo diphenyl guest and CA-POR. Cytotoxic effect of the NP nanoparticle through its SOG efficiency. Image used with permission from published work [61].

Yan et al. constructed an entire supramolecularly assembled nanoparticle mediated by host–guest interaction and used it to generate singlet oxygen for PDT [61]. The self-assembled structure was constructed based on a very simple idea of developing a supramolecular network between calixarene-tethered porphyrin (CA-POR), which contained the sensitizing unit, and macromolecular host; they used biviologen derivatives as the linker (Figure 22) that attached to the host through host–guest inclusion interaction to result in a self-assembled nanoparticle. The size and morphology of the photoactive NP matrix was controlled with the size of the linkers; with the right choice of linker (one example shown herein) the macromolecular sizes of the NPs were varied between 100 and 600 nm. Study of host–guest interaction based on spectroscopic Job’s plot analysis indicated that the cation–π interaction between the CA host and viologen guests is the primary sustaining force for the NP assembly. The utility of these nanoparticles as efficient PDT agents was demonstrated through their cytotoxicity toward HeLa cells. As shown in Figure 22, the nanoparticles showed good biocompatibility as negligible cell death occurred in the dark (black triangle), whereas a contrasting effect was observed when they were exposed to 633 nm visible light (red triangle), where more than 50% cell death was noticed in 60 min.

**Figure 23:**
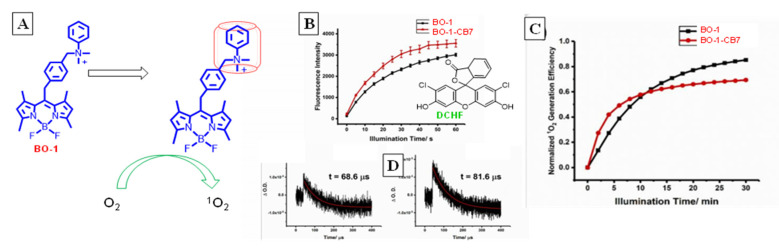
(**A**) Enhancement of SOG for BODIPY dye (BO-1) enhanced upon binding to CB7. (**B**) Fluorescence of DCHF increase used to monitor SOG for free and bound BO-1. (**C**) Photobleaching trend based on DCHF fluorescence indicating self-termination of the dye. (**D**) Triplet lifetimes of free (left) and complexed (right) BO-1. Image used with permission from published work [62].

A supramolecularly controlled on/off PS system was designed by Yuan et al. [62] that self-terminated after a PDT cycle, which is advantageous in minimizing dark toxicity of PSs (Figure 23). A BODIPY (BO-1) dye was used for this purpose, which showed enhanced SOG efficiency when included in CB7, compared to the free dye in aqueous solution. Strong binding was observed (1.46 × 10^6^ M^−1^) with CB7 for 1:1 complex. Laser flash photolysis measurements showed that the complexed BO-1 was long-lived (81.6 ms) in the triplet state compared to the free PS (68.6 ms). The prolonged lifetime of the triplet state enabled increased energy/electron transfer to O_2_, thus improving SOG, as evidenced using DCFH’s fluorescence as probe for monitoring singlet oxygen. The increased lifetime was presumably due to conferred rigidity, which reduces T_1_ → S_0_ ISC, or electronic quenching. Photobleaching of the dye was also studied by the researchers, wherein the CB7 complex underwent faster complete photobleaching (3 min) compared to the free dye (10 min). The increased photobleaching rate was attributed to both the higher ROS concentration in the medium for PS@CB7 as well as the increased oxidizability of bound dye as deduced from cyclic voltammetry, which showed decreased oxidation potential for the second step that was lowered by 51 mV.

**Figure 24:**
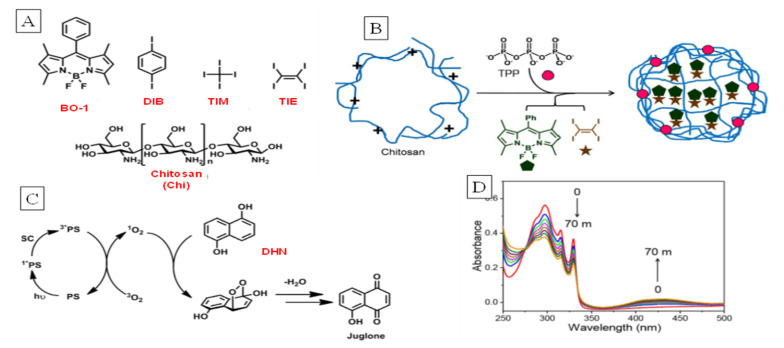
(**A**) Components of nanocomposite for SOG: BO-1, heavy-atom perturbers (DIB, TIM, TIE), and chitosan matrix (chi). (**B**) Depiction of the supramolecularly influenced SOG nanocomposite. (**C**) Mechanism of singlet oxygen generation and its reaction with DHN for monitoring. (**D**) Change in DHN and juglone mixture absorbance (signal from other species subtracted) with time for monitoring SOG. Image used with permission from published work [63].

A simple case of supramolecular confinement of the PS and a “heavy atom effect” promoter to enhance SOG efficiency was demonstrated by Naim et al. [63]. As formation of the triplet state of the PS is essential for SOG, improving the S_1_ → T_1_ rates should increase quantum yield (Φ_Δ_) of SOG. In this case, a BODIPY (BO-1) dye with low Φ_Δ_ (0.04) was converted into an efficient generator by supramolecular confinement, enforcing its co-occupation with iodine-containing hydrocarbons (Figure 24). Nanocomposites of BO-1 and tetraiodoethylene (TIE) were incorporated within matrices of the natural polymer chitosan using the ionic-gelation approach. Irradiation of the nanocomposite in the presence of dihydroxynaphthalene (SOG monitor) showed a clear increase in SOG efficiency as evidenced through UV/Vis spectral changes representing formation of the corresponding naphthoquinone (juglone) at 425 nm, which increased in intensity while that of DHN at 335 nm decreased. The enhancement of SOG was attributed to the well-known heavy atom effect of large atoms, in this case the proximally placed iodines in TIE, which would have increased the ISC rate of BODIPY through spin orbit coupling.

**Figure 25:**
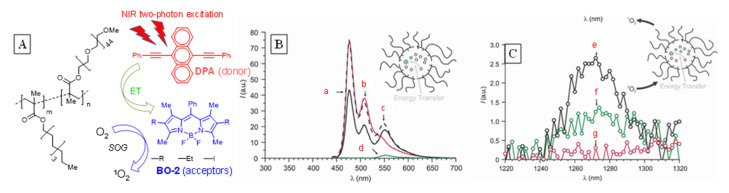
(**A**) Structures of donor, acceptor, and polymer used for construction of nanoreactor for intracellular singlet oxygen sensitization. (**B**) Emission spectra of nanoparticles containing donor and acceptor as emission of acceptor BO-2 observed upon excitation of donor DPA (**a**,**b**). Emission intensity of donor DPA corresponding to spectra (**c**,**d**), which decrease in presence of acceptor. (**C**) NIR emission of singlet oxygen generated when both donor and acceptor are present (**e**) vs. when only donor (**g**) or acceptor (**f**) is present. Image used with permission from published work [64].

The construction and use of a donor/acceptor supramolecular system for SOG activated by two-photon absorption for medical applications was reported by Raymo’s group (Figure 25) [64].

Two-photon chromophorism is less common but offers tremendous advantage for medical applications due to the tissue transparency, as the wavelength of light required for photoexcitation is significantly higher (typically above 600 nm). The donor/acceptor duo were chosen based on their spectral matching. The co-encapsulation of 9,10-disubstituted anthracene donor (DPA) along with BODIPYs (BO-2s) acceptor within the polymer matrix yielded a highly efficient singlet oxygen generator as monitored through its direct phosphorescence. The energy transfer from the donor to acceptor was evident through the dramatically enhanced fluorescence of the acceptor upon photoexcitation of the donor, corresponding to the decrease in intensity of the donor compared to its individual activity. This also correlated with the phosphorescence observed in the NIR region for singlet oxygen. The medical application of this system was demonstrated through their cytotoxicity toward the aggressive cervical cancer HeLa cells.

**Figure 26:**
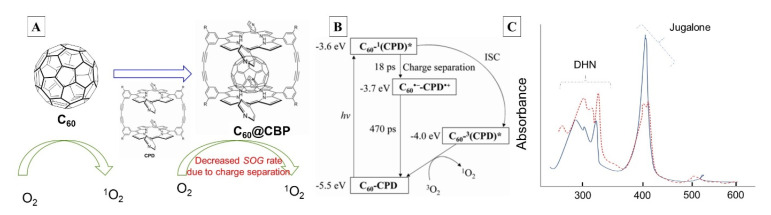
(**A**) Formation of supramolecular C_60_ dyad. (**B**) Mechanism of dyad function for SOG. (**C**) Monitoring of SOG through absorbance of DHN oxidation to juglone for free CBP (solid line, blue) vs. C_60_-CBP complex (broken line, red). Image used with permission from published work [73].

Photochemical dyads of covalently linked donor/acceptor units for influencing energy or electron transfer steps have been used for increasing the SOG efficiency of sensitizers [65,66,67,68]. Dyads are molecular constructs designed to perform specific functions by integrating two independently active units. The independent chemical units are integrated to produce a desired chemical function, such as an antenna chromophore that captures energy (photon) and efficiently transfers it (ET) to a metal complex capable of splitting water (2H_2_O → 2H_2_ + O_2_) [69]. Examples of dyads with C_60_ as one of the dyad components have been constructed as it improves triplet lifetimes of the sensitizing unit through increased ISC rates resulting from energy or electron transfer [66,70,71,72]. The supramolecular equivalent of such a strategy was explored by Ooyama et al. [73]. A porphyrin-containing molecular dimeric host was constructed, which was capable of encapsulating the C_60_ guest; with the porphyrin host being the sensitizer and C_60_ serving as electron/energy-transfer agent, it was expected that the resulting complex would also show increased singlet oxygen efficiency. However, to the contrary, the dimeric host sensitizer alone without the guest complexed showed a higher quantum yield of SOG (0.62) compared to the C_60_ complex (0.52). Though this was unexpected, the study of the photo-dynamics of the free host and the complex showed that photoexcitation resulted in a fully charge-separated state from the excited singlet state, instead of its transition to the triplet state (Figure 26B), which reduces its ability to act as a PS. The difference in SOG efficiencies was noticeable through absorbance changes in the DHN conversion to juglone (Figure 26C).

The use of cavitands in influencing oxidation reaction outcomes in terms of reactivity and selectivity was explored by Ramamurthy’s group [74]. Of particular value is the ene reactions studied within the octa acid host. The “ene reaction” of singlet oxygen with a reactant that contains multiple allylic hydrogen, such as 1-methyl cyclohexene, would result in at least three allylic hydroperoxides in homogeneous media. However, oxidation of methyl cyclohexene encapsulated within octa acid (OA) resulted in one isomeric product as the predominantly major product.

**Figure 27:**
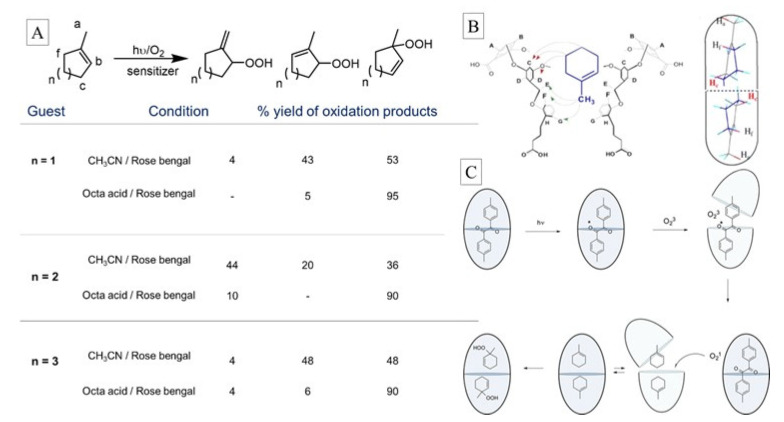
(**A**) Ene reactions and their product distribution in homogeneous media and within OA. (**B**) Structure of methyl cyclohexene with OA. (**C**) Mechanism of reaction selectivity deduced from supramolecular analysis. Image used with permission from published work [74].

Based on in-depth supramolecular analysis involving 2-D NMR experiments it was deduced that the regioselectivity in the reaction is due to the structurally specific inclusion of methyl cyclohexene within the OA cavity. The methyl group binds to the cavity facing inward toward the narrow end of OA, and OA is a dimeric host, as depicted in Figure 27. As the singlet oxygen enters the cavity, the first allylic hydrogen it is able to abstract is the c-hydrogen (reactant in Figure 27A and red hydrogens in Figure 27B). The resulting allyl radical rearranges to the tertiary radical, which couples with the oxygen radical to produce the tertiary allyl peroxide almost exclusively.

## 4. Conclusions

The instances of SOG systems outlined in this paper presented various approaches to influencing the photophysics of photosensitizers and energy-transfer events that influence the efficiency of this process. In all these cases, it was evident that the non-covalent interaction afforded by cavitands and other supramolecular components are very useful in manipulating the photo-dynamics of singlet oxygen generation. Be it enhancing the inherent quantum yield of SOG, or switching activity on/off on demand, or complete self-termination of the sensitizer itself after an SOG cycle for medical applications, the supramolecular approach provides a great degree of control to exploit the excited- and ground-state dynamics of the process. These are achieved through approaches such as sensitizer aggregation/disaggregation, manipulation of energy/electron transfer between donor/acceptor systems, perturbation of ISC rates with spin orbit coupling, dictating PS ambient oxygen interaction, etc. The various aspects of SOG are addressed without covalently modifying the sensitizer, which is convenient and efficient. The review also highlighted the creative ways with which chemists have tuned the efficiency of this reaction and used it for practical purposes. It is expected that the supramolecular approach to affecting SOG will find commercial use in the near future and more such creative uses of supramolecular chemistry will be explored.
